# Statistical treatment of Photoluminescence Quantum Yield Measurements

**DOI:** 10.1038/s41598-019-51718-4

**Published:** 2019-10-30

**Authors:** Felix Fries, Sebastian Reineke

**Affiliations:** Dresden Integrated Center for Applied Physics and Photonic Materials (IAPP) and Institute for Applied Physics, Technische Universität Dresden, 01187 Dresden, Germany

**Keywords:** Electronic properties and materials, Optical materials and structures, Characterization and analytical techniques

## Abstract

The photoluminescence quantum yield (PLQY) is an important measure of luminescent materials. Referring to the number of emitted photons per absorbed photons, it is an essential parameter that allows for primary classification of materials and further is a quantity that is of utmost importance for many detailed analyses of luminescent systems and processes. Determining the PLQY has been discussed in literature for many years and various methods are known. Absolute values can be measured directly using an appropriate setup. As this relies on the correct evaluation of photon-counts, it is a very sensitive method. Hence, systematic errors that can occur are discussed widely. However, of course those measurements also contain random uncertainties, which remain mainly unconsidered. The careful evaluation of both systematic and statistical errors of the PLQY is the only way to gain confidence in its absolute value. Here, we propose a way of evaluating the statistical uncertainty in absolute PLQY measurements. This relies on the combination of multiple measurements and the subsequent calculus of the weighted mean. The statistical uncertainty is then obtained as the standard deviation of the mean. This method not only quantifies the impact of statistical influences on the measurements, but also allows simple analysis of time-dependent systematic errors during the measurement and the identification of outliers.

## Introduction

In almost any application one can think of, the efficiency of the underlying processes is one of the key features. The efficiency is the ratio between effort put into the system and gain obtained from the system. From this general definition, many specific cases can be deduced. In electro-luminescent devices, such as organic^1^, perovskite^2^, or quantum dot LEDs^3^, maximizing the external quantum efficiency is often the driving motivation. Besides the careful engineering of the device architecture and electric performance, the efficiency directly depends on the inherent efficiency of the luminescent materials used^4^. The crucial factor is the ratio between emitted photons per molecular excitations. Due to the direct accessibility, this efficiency is normally quantified in photoluminescent experiments, rather than in electroluminescent ones, leading to the photoluminescence quantum yield (PLQY). Hence, the PLQY measures the ratio of emitted photons per absorbed photons. Therefore, when designing new materials, the PLQY is one key performance indicator – be it organic, perovskite, or quantum dots^5–7^. By its definition, the PLQY always takes values between 0 and 1. Exceptions are only meaningful if the system of investigation is known to undergo multi-exciton-generation processes such as singlet fission. In this specific case, values up to 2 are theoretically possible^8^.

Various ways to determine the PLQY are known. For solutions, a comparative method is frequently used that compares the luminescence of the molecule of interest to the one of a known standard^9,10^. Another way, which doesn’t rely on the comparison to a second material is the direct determination of the PLQY by measuring the number of absorbed photons and the number of emitted photons. This method has originally been introduced by de Mello *et al*. in 1997^11^, however the basic principle was already published two years earlier from the same group^12^. Nowadays, this technique is widely used (over 1100 citations) and is also tested for solutions^13^. In contrast to the comparative method, the direct determination of the PLQY has several advantages. Amongst others, it is a fast technique and does not rely on a known standard.

**Figure 1:**
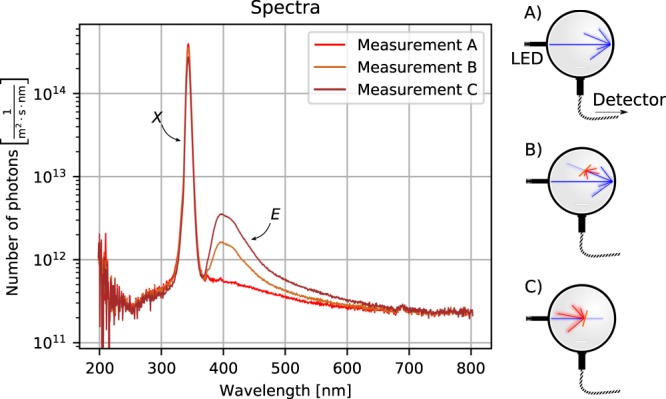
The three measurements needed for PLQY evaluation. On the left hand side the resulting spectra are shown and on the right hand side a scheme for each measurement constellation.

The setup consists of an excitation source, which can be a laser or LED. The light hits a luminescent sample, which is located within an integrating sphere. All the reflected, transmitted, or emitted light is then collected within the sphere and is subsequently detected. To determine the PLQY Φ, three measurements are necessary, as shown in Fig. 1. First, the excitation is led into the empty sphere to quantify the excitation intensity. Second, the sample is placed within the sphere, but not in the excitation beam. In that way, only diffuse reflected light from the sphere’s walls hits the sample. This step is necessary to determine the influence of repetitive excitation of the sample within the measurement time. Third, the sample has to be placed directly in the excitation beam.

Here, we will follow the original nomenclature, naming the three measurements A (empty sphere), B (indirect illumination of the sample), and C (direct illumination). If an intensity was measured, the data needs to be converted into a number of photons – a procedure that depends on the actual detector in use. Each spectrum taken consists of two parts: the residual excitation light and the emitted light. Both parts are integrated separately and are referred to as *X* (excitation) and *E* (emission), with a subscript denoting the respective measurement. With this, the absorption *A* and finally the PLQY Φ can be calculated^11^:

1 $$\begin{array}{c}A=(1-\frac{{X}_{{\rm{C}}}}{{X}_{{\rm{B}}}})\end{array}$$

2 $$\begin{array}{c}\Phi =\frac{{E}_{{\rm{C}}}-(1-A){E}_{{\rm{B}}}}{A\cdot {X}_{{\rm{A}}}}\end{array}$$

Even though this method is widely used and known for some time by now, it is not rare that the PLQY values for a given material differ strongly between different publications. For example, for the OLED emitter N,N′-diphenyl-N,N′-bis(1-naphtyl-phenyl)-1,1′-biphenyl-4,4′-diamine (NPB), the reported values range from 29–41%^14,15^. Additionally, a strong dependence of the PLQY can be observed for different sample preparations^16^. However, even under same conditions, it is known that many systematic influences have to be taken into account to get reliable results^17^. In contrast, to our knowledge no report on the treatment of the statistical uncertainty of PLQY measurements is reported so far. Here, we propose a method, doing multiple measurements and using weighted means for the evaluation. In this way, a very low statistical uncertainty can be achieved and additionally further insights in the influence of single measurements are possible.

## Sources of Statistical Errors

The PLQY, not different to any other experimentally determined quantity, has a measurement uncertainty. This uncertainty has to be split into two contributions: the systematic and the statistical uncertainty, ${\Delta \Phi }_{{\rm{sys}}},\,{\Delta \Phi }_{{\rm{stat}}}$ respectively. Quantifying both values is of very basic importance to be able to estimate the correctness of the resulting value. Sources of systematic errors are amongst others the excitation angle, the sensitivity of the detection instrument, and the responsivity of the sphere^12,15,17,18^. The angle of incidence should be as close to 90° as possible to minimize parasitic internal reflections. The detector can be either a calibrated spectrometer or a well-characterized photodiode^18^. The responsivity of the integrating sphere is also a very important parameter as each photon hypothetically is reflected infinite times on the sphere’s wall, and thus suffers strongly of wavelength-dependent absorption. This changes the integrated values for excitation and emission (${X}_{i},{E}_{i}$) differently. Even assuming that each contribution differs by a constant value, it is obvious in Eqs (1) and (2) that a constant additive factor doesn’t cancel out and thus changes the final result.

Having the measurement system set up as correct as possible and accounted for all known systematic errors or uncertainties, there is still the influence of the statistical or random error. Each measurement suffers random deviations. Sources for such deviations are counting errors of the spectrometer, electric noise in any wire or detector, intensity variations of the light source, intensity fluctuations of the emission, and overlap of excitation and emission. The first two points will result in a slightly shifted noise level or a change of the spectral distribution of the latter. As mentioned in the section above this can be compared to a constant additive term to *X*_*i*_ and thus will change the resulting PLQY. Intensity variations of the light source will obviously depend on its quality. However, it can never be suppressed completely. Now, it lies in the nature of this measurement technique that measurements obtained at different times have to be compared (A-, B,- C-measurement). Strictly spoken, any fluctuation in the excitation density prohibits this comparison. Still, these influences can be mapped very nicely by just doing multiple measurements of each type (A, B, C). This extends the relevant time window and thus collects way more fluctuation. Additionally, specific influences on the resulting value can be identified, as will be discussed at the end of this article. The overlap of excitation and emission regime can also be attributed to the systematic deviations^17^. Still, even if the spectra are nicely separated, it is ab initio not clear, where to set the borderline between the spectral ranges of absorption and emission. Hence, varying this parameter for each data set taken, can introduce a random like behaviour in the measurement uncertainty. That way one set of data leads to one PLQY and a correlating uncertainty of ${\Phi }_{i}\pm {\Delta \Phi }_{{\rm{s}}{\rm{t}}{\rm{a}}{\rm{t}},{i}}$.

## Multiple Measurements and Statistical Evaluation of the PLQY

As a matter of fact, the way of determining the PLQY as is done here, allows for a very good statistical treatment. To obtain one value of PLQY, three independent measurements are necessary. The next step is as simple as powerful: if each measurement is taken several times, there should be no difference amongst them, except for the random variations discussed above. As a direct consequence, no definite assignment of measurements is given. In contrast, from a physical point of view, each combination of the acquired A-, B-, and C-measurement should result in the very same PLQY. This leads to the fact that there will be *n*^3^ PLQY values, if each measurement is taken $n$ times. Assuming for example an average integration time of 2 s per spectrum, within one minute of measurement time, 30 spectra can be taken leading to 1000 PLQY values (with $n=10$). Note that the highest output compared to the measurement effort will always be for the same number of all three measurements, i.e. ${n}_{{\rm{A}}}={n}_{{\rm{B}}}={n}_{{\rm{C}}}$.

The final value then results as the mean of the single values. However, as mentioned above, each single value already has a statistical uncertainty. Therefore, a reasonable treatment of the data appears to be the calculation of the weighted mean^19^. Here, each value Φ_*i*_ is weighted with a weight *w*_*i*_, which correlates to the inverse of its variance ${\sigma }_{i}^{2}$ ^20^:

3 $$\begin{array}{c}{w}_{i}=\frac{1}{{\sigma }_{i}^{2}}.\end{array}$$

The weighted mean $\bar{\Phi }$ is given as:

4 $$\begin{array}{c}\bar{\Phi }=\frac{{\sum }_{i}{w}_{i}\cdot {\Phi }_{i}}{{\sum }_{i}{w}_{i}}.\end{array}$$

The corresponding standard deviation *σ* of the sample is then given as:

5 $$\begin{array}{c}\sigma =\sqrt{\frac{{\sum }_{i}{w}_{i}\cdot {({\Phi }_{i}-\bar{\Phi })}^{2}}{{\sum }_{i}{w}_{i}\cdot (1-\frac{1}{n})}}.\end{array}$$

To come from a sample standard deviation to the mean’s standard deviation, one has to divide by the square root of the number of measurements:

6 $$\begin{array}{c}\bar{\sigma }=\sqrt{\frac{{\sum }_{i}{w}_{i}\cdot {({\Phi }_{i}-\bar{\Phi })}^{2}}{{\sum }_{i}{w}_{i}\cdot (n-1)}}.\end{array}$$

The factor $(1-\frac{1}{n})$ in the denominator of Eq. (5) was not introduced by Price *et al*.^19^, but is a common correction, necessary to obtain an unbiased estimator. Sometimes this is referred to as Bessel Correction^21^. For comparison with unweighted calculations, all weights are set to an equal value ${w}_{i}=w={\rm{const}}.$. The summation in the denominator will thus give $\mathop{\sum }\limits_{i}^{n}{w}_{i}=w\cdot \mathop{\sum }\limits_{i}^{n}1=w\cdot n$. Now $w$ can be cancelled and the upper equations result in the same expression as their unweighted counterpart:

$$\begin{array}{c}{\sigma }_{{w}_{i}=w}=\sqrt{\frac{{\sum }_{i}{({\Phi }_{i}-\bar{\Phi })}^{2}}{n-1}},\,{\bar{\sigma }}_{{w}_{i}=w}=\sqrt{\frac{{\sum }_{i}{({\Phi }_{i}-\bar{\Phi })}^{2}}{n\cdot (n-1)}}\,.\end{array}$$

Especially when the excitation and the emission are nicely separated in the spectra, all the *σ*_*i*_ will be very similar. It’s important to note, that *n* in Eqs (5) and (6) refers to the number of measurements and not of different PLQY values.

## Discussion

**Figure 2:**
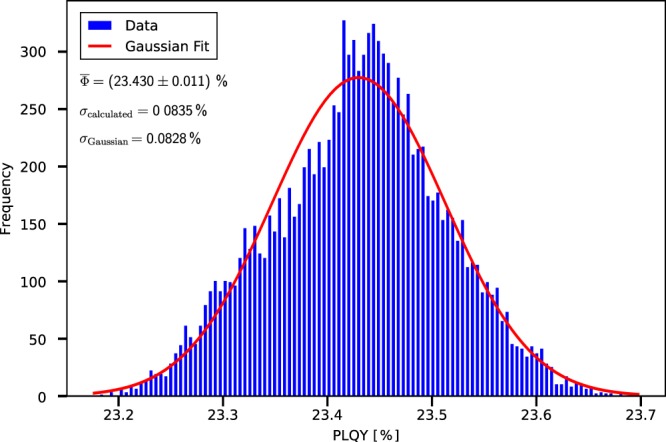
The statistical distribution of PLQY values for an example device. The Gaussian fit is a guide to the eye. The PLQY is given in the form $\bar{\varPhi }=({\varPhi }_{weighted}\pm {\bar{\sigma }}_{statistical})$.

Assuming the *N* different PLQY values were uncorrelated, a Gaussian distribution around the mean value $\bar{\Phi }$ would be expected. However, as they result from only $n < N$ independent measurements, they are correlated. Therefore, a Gaussian fit can only be done as a guide to the eye, but cannot serve as a permissible way of evaluation. However, if any systematic variations can be excluded, a Gaussian curve may resemble the distribution of the data quite well. In Fig. 2 such example is shown. For this data set, Eq. (3) gives $\sigma =0.0835 \%$, which nearly equals the one of the Gaussian fit of ${\sigma }_{{\rm{Gaussian}}}=0.0828\, \%$. With ${n}_{{\rm{A}}}={n}_{{\rm{B}}}={n}_{{\rm{C}}}=23\Rightarrow n=69$ this results in a very low $\bar{\sigma }=0.011\, \%$.

**Figure 3:**
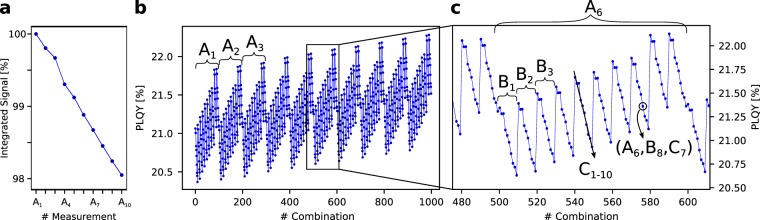
Plotting the resulting PLQY for each evaluation step separately gives a repetitive pattern. (**a**) Shows the integrated values of each A-measurement. They are normalized to the first value. In (**b**) each block of data refers to one A-measurement combined with every B-, and C-measurement. Each of these blocks contains a substructure (**c**), where each B-measurement is combined with all the C-measurements. The influence of the C-measurements can be derived from the substructure therein. In this example, a systematic error in the form of an intensity decay of the light-source was introduced on purpose. This leads to the increase of the PLQY with increasing A-, and B- measurement, and a decreasing C-measurement.

Besides the estimation of the statistical uncertainty of Φ, this evaluation technique allows a detailed identification of systematic errors made during the measurement. Plotting the PLQY of each combination of data files, time-dependent trends can easily be detected. Such effects can e.g. be a non-stable light source, degradation of the sample, or unstable environmental conditions. Figure 3 shows an example, where on purpose the PLQY measurement was started, even though the light source was not yet in a thermal stable state but still losing intensity while heating up. This leads to a continuous decrease of intensity with each measurement, which can be seen in the integrated values in Fig. 3a. Here, only the A-measurements are shown, but the B- and C-measurements show the same trend. The total number of measurements is ${n}_{{\rm{A}}}={n}_{{\rm{B}}}={n}_{{\rm{C}}}=10$. The order of evaluation in this example is: the first A-measurement (*A*_1_) and the first B-measurement (*B*_1_) are combined with every C-measurement one after the other (${C}_{1-10}$). Subsequently, the second B-measurement is taken, and again all the C-measurement. This is continued until each combination has been evaluated. In the end, this leads to the repetitive pattern in Fig. 3b,c, where the major blocks refer to combinations with different A-, the minor blocks to B-, and the substructure to the different C-measurements. It is visible, that Φ is systematically increasing from one A-measurement to another. As the only relevant influence on Φ here is *X*_*A*_ in the denominator of Eq. (2), the decreasing intensity of the excitation LED leads to an increasing PLQY. The trend for B, and C can be explained in a similar manner. However, here the main influence is due to the change of the calculated absorption. In this example, it is decreasing for the B-measurements, and increasing for the C-measurements.

Having listed the different PLQY values has yet another beneficial effect. The robustness of the method can directly be determined by comparing the fluctuations in the original spectra with the resulting changes in the PLQY. Taking the points of Fig. 3 again, it can be deduced, that the A-, B-, and C-measurement lead to a PLQY variation of 2.0%, 3.5%, and 3.3%, respectively. On the other hand, the measured spectra can directly be integrated to have a measure for the fluctuation in the original data. Here, the three measurements suffer intensity changes of 2.0% (A), 1.1% (B), and 0.8% (C). This teaches that the strongest relative influence on the statistical uncertainty has the C-measurement, whereas the A-measurement has the lowest impact. Having this in mind, the condition of having the same number of measurements (${n}_{{\rm{A}}}={n}_{{\rm{B}}}={n}_{{\rm{C}}}$), should always be fulfilled to not over- or underestimate the statistical error.

For a given stable setup, the resulting statistical error should always be in a similar range. This means that obtaining unexpected large values can be a direct indicator for more severe mistakes.

Of course, the statistical error can also be quantified by calculating the mean and the standard deviation of each ${X}_{i}$ and ${E}_{i}$ separately. The uncertainty of the resulting $\Phi$ would then be obtained using the propagation of the error of each. However, this way is neither faster, simpler, nor more trustworthy. In contrast, it does not provide any further information about systematic trends in the data as discussed in the paragraphs above.

## Summary

Based on the method presented already in 1992 by de Mello *et al*. for measuring the absolute value of the PLQY, we discussed a way to quantify the statistical uncertainty. Therefore, each type of the three individual spectra (A, B, C) has to be measured several times. A random error resulting from the non-defined separation between excitation and emission part of the spectrum is calculated for each spectrum. If this influence is small, the weighted calculation reduces to the unweighted methods known from any statistics textbook. In either case, statistical influences on the measurement are nicely mapped in the distribution of PLQY values. Besides estimation of the statistical uncertainty of the measured value, this method allows for a detailed analysis of the data and helps to identify false measurements. Additionally, the influence of each measurement type on the resulting PLQY has been estimated. It turns out, that the C-measurement has the strongest impact, whereas the A-measurement has the lowest.

It is very important to have in mind that this whole procedure only and exclusively treats the random influences on the resulting PLQY. Systematic errors need a treatment on their own and often result in substantially larger values. Additionally, when basic mistakes are present either in the experimental setup or during the measurement, a small statistical error cannot be taken as a warrant for correct results. Still, knowing that the statistical uncertainty of a setup is small helps to identify other sources of errors.

## Methods

The setup consists of an UV-LED of 340 nm (Thorlabs, M340L4) as excitation, where of course LEDs of other centre wavelength should be chosen, with respect to the emitter of investigation. The light is collimated, filtered with a 340 nm bandpass filter, and directed into an integrating sphere of Labsphere. The diameter of the sphere is 6 inch, where the area of all ports make less than 0.6‰ of the sphere’s surface. The detection is performed using a calibrated spectrometer (Instrument Systems, CAS 140CT) with a detection range from 200–800 nm. The output data comes in $\frac{{\rm{W}}}{{{\rm{m}}}^{2}\cdot {\rm{nm}}}$ – a spectral power density per area. Multiplying with the wavelength and dividing by $h\cdot c$ (Planck’s constant times speed of light) gives a spectral photon density per area and time in $\frac{1\cdot {10}^{-9}}{{{\rm{m}}}^{2}\cdot {\rm{nm}}\cdot {\rm{s}}}$.

The response of the sphere was determined by comparing the directly measured spectrum of a white-light source with the one obtained at the exit-port of the integrating sphere. In the UV-wavelength regime, the same was done with UV-LEDs. Those response curves were combined to one, covering the complete detection range.

The presented experimental data are available from the corresponding authors.
